# Osteopontin attenuates early brain injury through regulating autophagy‐apoptosis interaction after subarachnoid hemorrhage in rats

**DOI:** 10.1111/cns.13199

**Published:** 2019-08-22

**Authors:** Cheng‐Mei Sun, Budbazar Enkhjargal, Cesar Reis, Ke‐Ren Zhou, Zhi‐Yi Xie, Ling‐Yun Wu, Tong‐Yu Zhang, Qi‐Quan Zhu, Ji‐Ping Tang, Xiao‐Dan Jiang, John H. Zhang

**Affiliations:** ^1^ The National Key Clinical Specialty, The Engineering Technology Research Center of Education Ministry of China, Guangdong Provincial Key Laboratory on Brain Function Repair and Regeneration, Department of Neurosurgery Zhujiang Hospital, Southern Medical University Guangzhou China; ^2^ Department of Physiology and Pharmacology Loma Linda University Loma Linda CA USA

**Keywords:** apoptosis, autophagy, early brain injury, osteopontin, subarachnoid hemorrhage

## Abstract

**Aim:**

To determine the effect of osteopontin (OPN) on autophagy and autophagy‐apoptosis interactions after SAH.

**Methods:**

The endovascular perforation model of SAH or sham surgery was performed in a total of 86 Sprague‐Dawley male rats. The temporal expressions of endogenous OPN and autophagy‐related proteins (Beclin 1, ATG5, LC3 II to I ratio) were measured in sham and SAH rats at different time points (3, 6, 12, 24, and 72 hours). Rats were randomly divided into three groups: Sham, SAH + Vehicle (PBS, phosphate‐buffered saline), and SAH + rOPN (5 μg/rat recombinant OPN). Neurobehavioral tests were performed 24 hours after SAH, followed by the collection of brain samples for assessment of autophagy and apoptosis proteins. These tests assessed whether an autophagy‐apoptosis relationship existed on the histological level in the brain.

**Results:**

Endogenous OPN and autophagy‐related proteins all increased after SAH. rOPN administration improved neurological dysfunction, increased the expression of autophagy‐related proteins (Beclin 1, ATG5, LC3 II to I ratio) and antiapoptotic protein Bcl‐2, while decreasing the expression of proapoptotic proteins (cleaved Caspase‐3 and Bax). rOPN also regulated autophagy‐apoptosis interactions 24 hours after SAH.

**Conclusion:**

rOPN attenuates early brain injury and inhibits neuronal apoptosis by activating autophagy and regulating autophagy‐apoptosis interactions.

## 1 INTRODUCTION

Aneurysmal subarachnoid hemorrhage (SAH) remains to be one of the most devastating forms of stroke with high mortality and disability rates throughout the world.^1^ In recent years, early brain injury (EBI) has been reported as a primary cause of mortality in SAH patients.^2^, ^3^ The initiation of many important pathological mechanisms that happens within minutes following aneurysm rupture,^4^ requires ongoing research to improve the understanding of the pathophysiological events occurred. 5

Among the various pathological processes contributing to EBI, neuronal apoptosis has been reported to be an essential process that might explain the severe impact of SAH on short‐term and long‐term outcomes.^6^ Meanwhile, as a highly conserved biological phenomenon to degrade intracellular components and help maintain cellular homeostasis,^7^ autophagy activation after SAH has been shown to have neuroprotective effects. Several studies have reported that autophagy enhancing drugs can reduce apoptosis, while autophagy inhibitors aggregate cell apoptosis after SAH induction.^8^, ^9^, ^10^ Autophagy is an important protective mechanism against apoptosis in ischemic cell injury.^11^ Activation of autophagy protects neuronal apoptosis in EBI after SAH.^12^ However, few studies are investigating the relationship between autophagy and apoptosis in EBI after SAH on the molecular and cellular level.

Osteopontin (OPN) is a secreted extracellular matrix glycoprotein with roles in a variety of physiological and pathological processes, including cerebral vascular remodeling, cell migration, and antiapoptotic processes.^13^ In previous studies, administration of recombinant OPN (rOPN) has shown promising neuroprotective effects^14^ such as stabilizing vascular smooth muscle cell phenotype after subarachnoid hemorrhage,^15^ and attenuating inflammation after intracerebral hemorrhage.^16^ Interestingly, recent studies showed that OPN was a potential enhancer of autophagy in an in‐vitro model of abdominal aorta aneurysm^17^ and human hepatocellular carcinoma.^18^ This indicates that one of the important mechanisms underlying OPN's neuroprotective effects after SAH could be its antiapoptotic effect through autophagy activation. No previous research has investigated the influence of OPN on autophagy or autophagy‐apoptosis interactions in SAH models. In the present study, we aimed to determine the regulatory role of OPN in autophagy modulation and autophagy‐apoptosis interaction to understand the neuroprotective effects of OPN treatment in SAH better.

## 2 MATERIALS AND METHODS

### 2.1 Animals and SAH model

A total of 86 adult male Sprague‐Dawley rats (290‐330 g, Harlan, Indianapolis, IN, USA) were housed in a room with constant temperature (25°C), humidity control, 12 hours/12 hours light/dark cycle, and with free access to food and water. All the experimental procedures were performed following a corresponding study protocol approved by the Institutional Animal Care and Use Committee (IACUC) of Loma Linda University, and under the NIH Guide for the Care and Use of Laboratory Animals.

The endovascular perforation model of SAH was conducted following previously described procedures.^19^ All rats were randomly assigned to experimental groups. Briefly, rats were intubated in deep anesthesia and kept on a rodent ventilator (Harvard Apparatus) during surgery with 3% isoflurane in 65/35% medical air/oxygen to maintain anesthesia. The left external and internal carotid artery were exposed, and a 4‐0 monofilament nylon suture was inserted into the left internal carotid artery through the dissociated external carotid artery until resistance was felt. The suture was advanced 3 mm further to perforate the bifurcation of the anterior and middle cerebral artery followed by immediate withdrawal. Sham rats underwent the exact same procedures except for the perforation. The incision sutured and rats were housed individually in heated cages following recovery from anesthesia. At 24 hours after sham or SAH surgery, SAH score was evaluated by an independent observer as previously described^20^: the total score (maximum SAH grade = 18) was calculated as the sum of six sub‐scores based on six corresponding predetermined areas. Animals with mild SAH (SAH grade ≤ 8) were excluded from the current study.

### 2.2 Experimental design

#### 2.2.1 Experiment 1: Time‐course study

To determine the temporal expression of endogenous OPN and autophagy‐related proteins (Beclin 1, ATG 5 and LC3) after SAH, 36 rats were divided into six groups: Sham, SAH 3 hours, SAH 6 hours, SAH 12 hours, SAH 24 hours, and SAH 72 hours (n = 6 per group). Western blot was performed to determine the expression of the four target proteins.

#### 2.2.2 Experiment 2: Localization study

To determine the distribution of endogenous OPN and autophagy‐initiating protein Beclin1 in different types of cells after SAH, three rats were used for double immunohistochemistry staining, performed at the time point after SAH when the peak expression of the two target proteins occurs.

#### 2.2.3 Experiment 3: Outcome study

To evaluate the possible beneficial effects of rOPN after SAH, 27 rats were randomly divided into three groups (n = 9 per group): Sham, SAH + Vehicle (30 μL PBS), and SAH + rOPN (5 μg/rat recombinant Osteopontin in 30 μL PBS, 6359‐OP‐050, R&D Systems). rOPN dosage was chosen based on our previous research.^21^ The nine rats per group included six rats for neurological tests and Western blot, and 3 for immunofluorescence staining. Vehicle or rOPN was delivered via the intranasal route 1 hour after SAH induction. Rats under isoflurane anesthesia were placed in a supine position and PBS or rOPN dissolved in PBS was administered alternately into the left and right nares, 5 μL each time with an interval of 2 minutes between each administration. A total volume of 30 μL was administered intranasally to each animal.

### 2.3 Neurobehavior assessment at 24 hours after SAH

Neurobehavior was evaluated at 24 hours after SAH induction using the previously described modified Garcia scoring system and beam balance test in a blinded fashion.^22^ In modified Garcia test,^23^ six parameters were tested, which allowed a total score of 18. Higher scores indicated a greater functional assessment of neurological outcome. These six tests included: spontaneous activity, symmetry in the movement of all four limbs, forepaw outstretching, climbing, body proprioception, and response to vibrissae touch. The beam balance test^24^, ^25^ consisted of rats walking on a 15‐mm wide wooden beam for 1 minutes. The mean score was calculated based on three consecutive trials that were scored from 0 to 4 according to the rats' walking ability.

### 2.4 Western blot analysis

Western blot tests were performed as previously reported.^26^, ^27^ Briefly, the whole left hemispheres were isolated and collected 24 hours after SAH. After protein extraction, equal amounts of protein samples were loaded onto each lane of SDS‐PAGE gel. After electrophoresis, the protein samples were transferred onto a nitrocellulose membrane for blocking and incubation at 4°C overnight with the following primary antibodies: anti‐Osteopontin (1:1000, sc‐21742), and anti‐β‐actin (1:5000, I‐19) from Santa Cruz Biotechnology Inc; anti‐Beclin 1 (1:1000, NB500‐249), anti‐ATG 5 (1:500, NB110‐53818), anti‐LC3 (1:5000, NB600‐1384), anti‐Caspase‐3 (1:200, NB100‐56708), and anti‐Bax (1:500, NBP1‐28566) from Novus Biologicals; anti‐Bcl‐2 (1:1000, ab59348) and anti‐SQSTM1/p62 (1:5000, ab56416) from Abcam. Secondary antibodies used were anti‐mouse (1:5000, sc‐2031), anti‐goat (1:5000, sc‐2354) from Santa Cruz Biotechnology Inc, and anti‐rabbit (1:5000, 2839792) from EMD Millipore Corporation.

### 2.5 Double immunofluorescence staining and TUNEL staining

Rats were sacrificed at 24 hours after SAH induction. A series of 8 μm‐thick frozen brain tissue slices were prepared. Double immunofluorescence staining was performed as previously described.^28^, ^29^ The primary antibodies used were anti‐GFAP (1:500, ab53554), anti‐NeuN (1:500, ab177487), and anti‐IBA 1 (1:500, ab107159) from Abcam; anti‐Osteopontin (1:100, sc‐21742) from Santa Cruz Biotechnology Inc; anti‐Beclin 1 (1:100, NB500‐249), and anti‐Caspase‐3 (1:50, NB100‐56708) from Novus Biologicals. Corresponding secondary antibodies were purchased from Jackson Immunoresearch and were applied at a concentration of 1:500. Nuclei were counterstained with DAPI (blue). After staining, the sections were visualized and photographed with a fluorescence microscope (Leica Microsystems).

TUNEL staining was performed with an in situ cell death detection kit (TUNEL) following the manufacturer's instructions (11684795910, from Roche Diagnostics). Nuclei were counterstained with DAPI (blue). Three rat brains per group (six sections per brain) were used for quantification analysis. Images of TUNEL‐positive cells in designated locations were captured at ×200 magnification by an independent observer with a fluorescence microscope (Leica Microsystems). Image J software (Image J 1.4, NIH) was used for cell counting. Also the data were presented as the average number of TUNEL‐positive cells per square millimeter (cell/mm^2^).

### 2.6 Statistical analysis

All data were expressed as mean ± SD (standard deviation). After the normality test, one‐way ANOVA of the mean values followed by Tukey's post‐hoc test was performed for multiple groups. A Kruskal‐Wallis test was used for Garcia scores, beam balance test, and SAH grading scores. The analyses were performed using SPSS version 24.0 (IBM Corp.). Statistical significance was defined as *P* < .05.

## 3 RESULTS

### 3.1 Mortality and SAH grades

**Figure 1:**
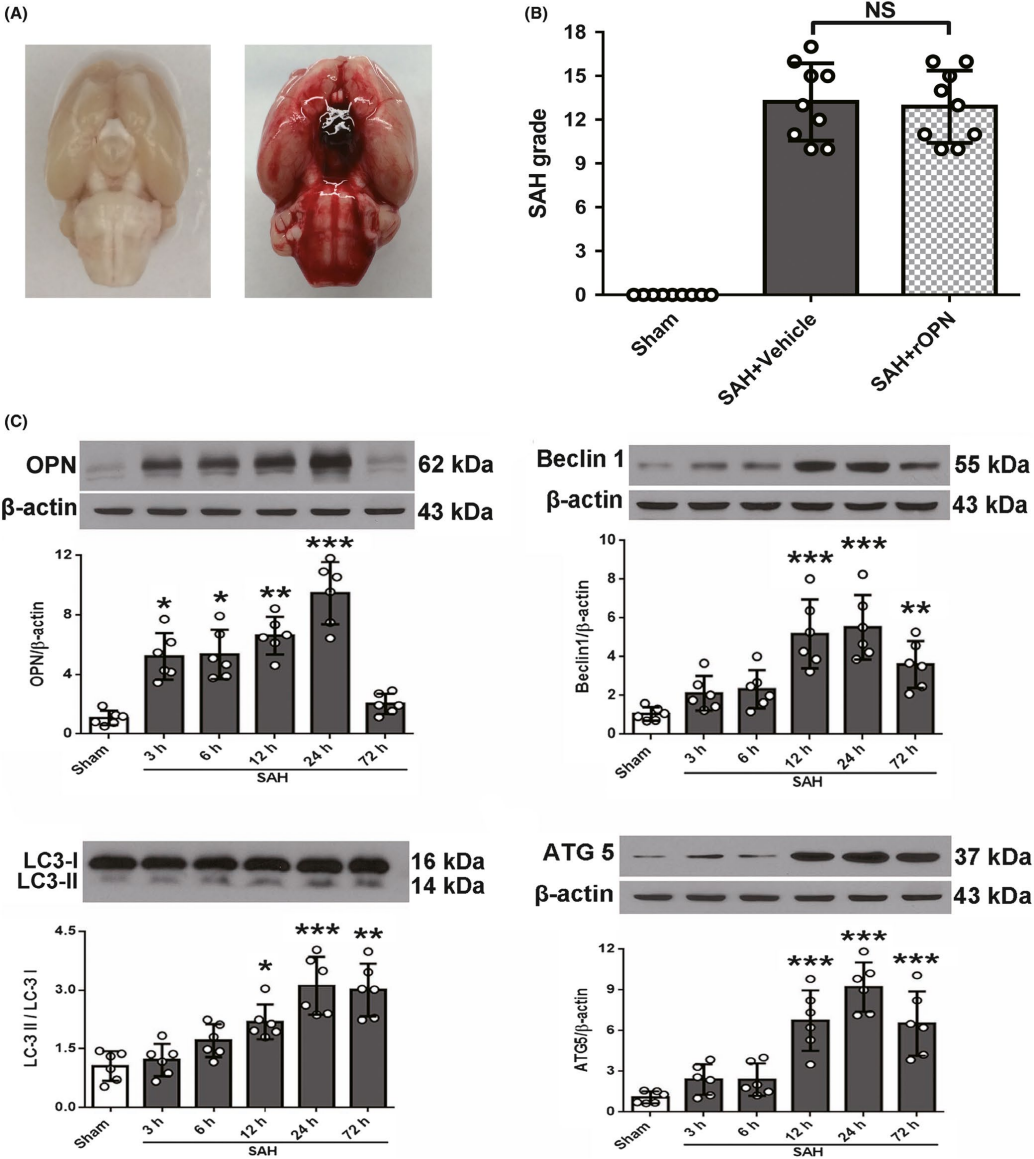
Subarachnoid hemorrhage (SAH) grades and temporal expression of endogenous osteopontin (OPN) and autophagy‐related proteins in rat brain after SAH. A, Representative brain images of Sham and SAH rats. B, Summary of SAH grading scores of all groups. Sample size is 27, n = 9 per group. Data were analyzed using Kruskal‐Wallis test, *χ* ^2^ = 18.183. C, Representative Western blot images and quantitative analyses of OPN, Beclin 1, LC3, and ATG 5 from the left hemisphere of rat brains at different time points after SAH. Sample size is 36, n = 6 per group. Data were presented as mean ± SD. *F* = 28.45 for OPN, *F* = 12.37 for Beclin 1, *F* = 18.88 for LC3, *F* = 22.14 for ATG5. *P < .05, ** P < .01, ***P < .001 vs Sham group. SAH, subarachnoid hemorrhage; Vehicle, phosphate‐buffered saline; rOPN, recombinant OPN. NS, not significant

A total of 86 rats were used: 15 rats were sham, 71 rats underwent SAH induction. Seven rats were excluded from the study due to mild SAH with grade ≤ 8 (Table 1). The mortality (calculated after exclusion of low‐grade rats) of SAH rats was 20.31% (13/64). No rats died in the sham group. Blood clots were mainly seen around the circle of Willis and ventral brain stem after SAH induction (Figure 1A). The average SAH grades showed no significant differences between SAH + Vehicle group and SAH + rOPN group (Figure 1B).

**Table 1:** Summary of animal usage, mortality, and exclusion

Experimental study groups	Mortality	Exclusion
Experiment 1. Time‐course study		
Sham	0 (0/6)	0
SAH (3, 6, 12, 24, 72 h)	18.92% (7/37)	4
Experiment 2. Localization study		
SAH (24 h)	25% (1/4)	0
Experiment 3. Outcome study		
Sham	0 (0/9)	0
SAH + Vehicle (PBS)	18.18% (2/11)	2
SAH + rOPN	25% (3/12)	1
Total		
Sham	0 (0/15)	0
SAH	20.31% (13/64)	7

Abbreviations: PBS, phosphate‐buffered saline; rOPN, recombinant osteopontin; SAH, subarachnoid hemorrhage.

### 3.2 Temporal expression of endogenous OPN and autophagy‐related proteins after SAH

Western blot was performed to determine the expression of endogenous OPN and autophagy‐related proteins (Beclin 1, ATG 5 and LC3) at 3, 6, 12, 24, and 72 hours on the left hemispheres of rats' brains after SAH. Results showed that endogenous OPN & LC3‐II to I gradually increased from 3 hours after SAH induction, while Beclin 1 and ATG 5 got a sudden increase starting from 12 hours. All the autophagy‐related proteins and OPN peaked at 24 hours. However, OPN and Beclin 1 level started to drop at 72 hours after SAH while the expression of ATG 5 and LC3 II/I remained stable till 72 hours after SAH (Figure 1C).

### 3.3 Expression of endogenous OPN and Beclin 1 in different cell types after SAH

**Figure 2:**
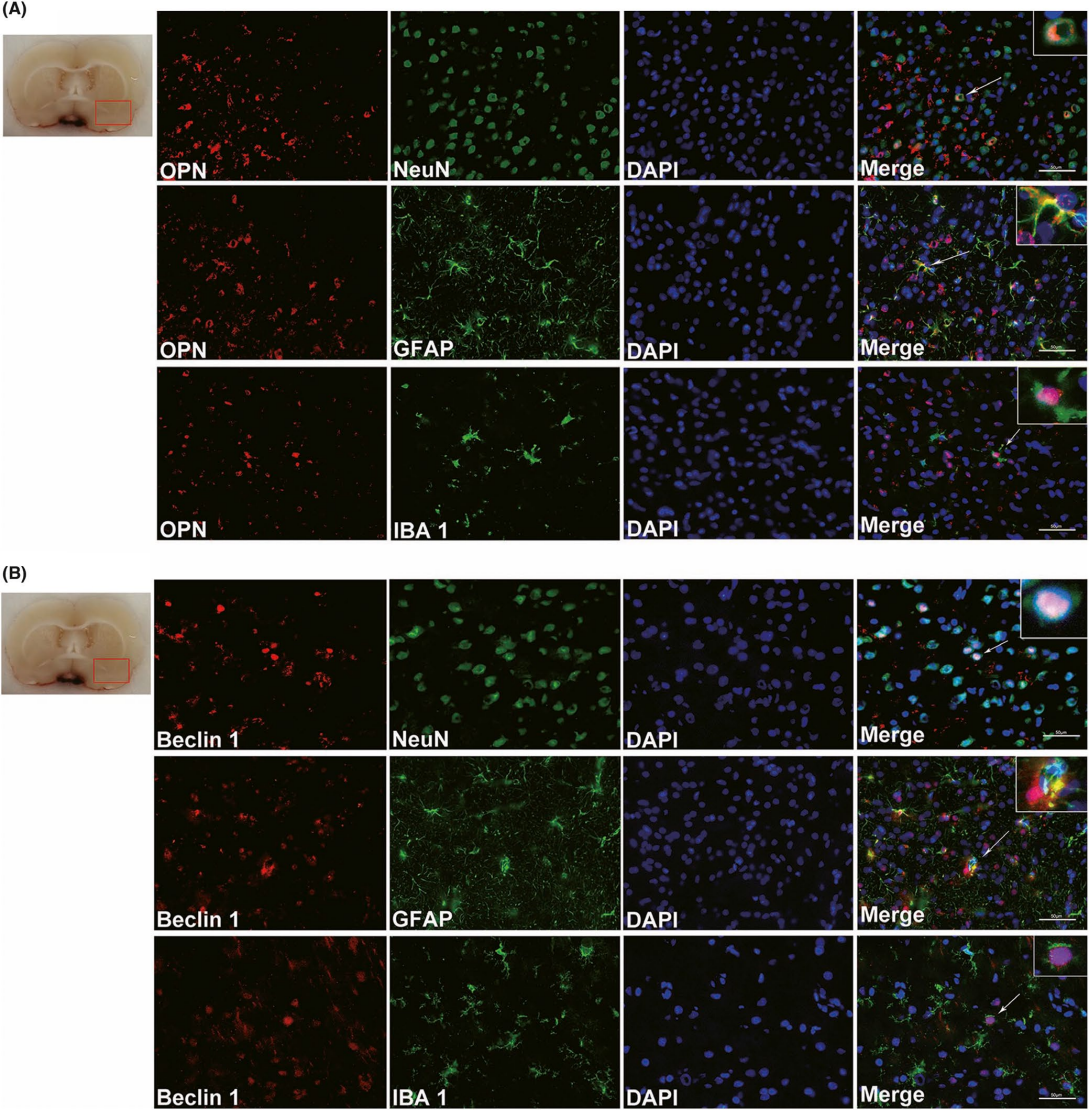
Expression of endogenous osteopontin (OPN) and Beclin 1 in different cell types of rat brain at 24 h after SAH. Double immunofluorescence staining for endogenous OPN (A) or Beclin 1 (B) in neurons (NeuN, green), astrocytes (GFAP, green) and microglia (IBA‐1, green) in the left basal cortex of rat brain at 24 h after SAH. Nuclei are stained with DAPI (blue). The red box in the brain slice image indicates the area observed. n = 3 per group. Scale bar = 50 μm

Since the expression of endogenous OPN and Beclin 1 both peaked at 24 hours after SAH, double immunohistochemistry was performed on SAH rats at 24 hours to show localization of OPN and Beclin 1 in neurons (NeuN, green), astrocytes (GFAP, green), and microglia (IBA 1, green). Results demonstrated that both endogenous OPN (Figure 2A) and Beclin 1 (Figure 2B) were expressed in all three types of cells after SAH, and both were mainly expressed in neurons, indicating their potential participation in neuronal survival and function.

### 3.4 Intranasal administration of rat rOPN ameliorated neurological deficits at 24 h after SAH

**Figure 3:**
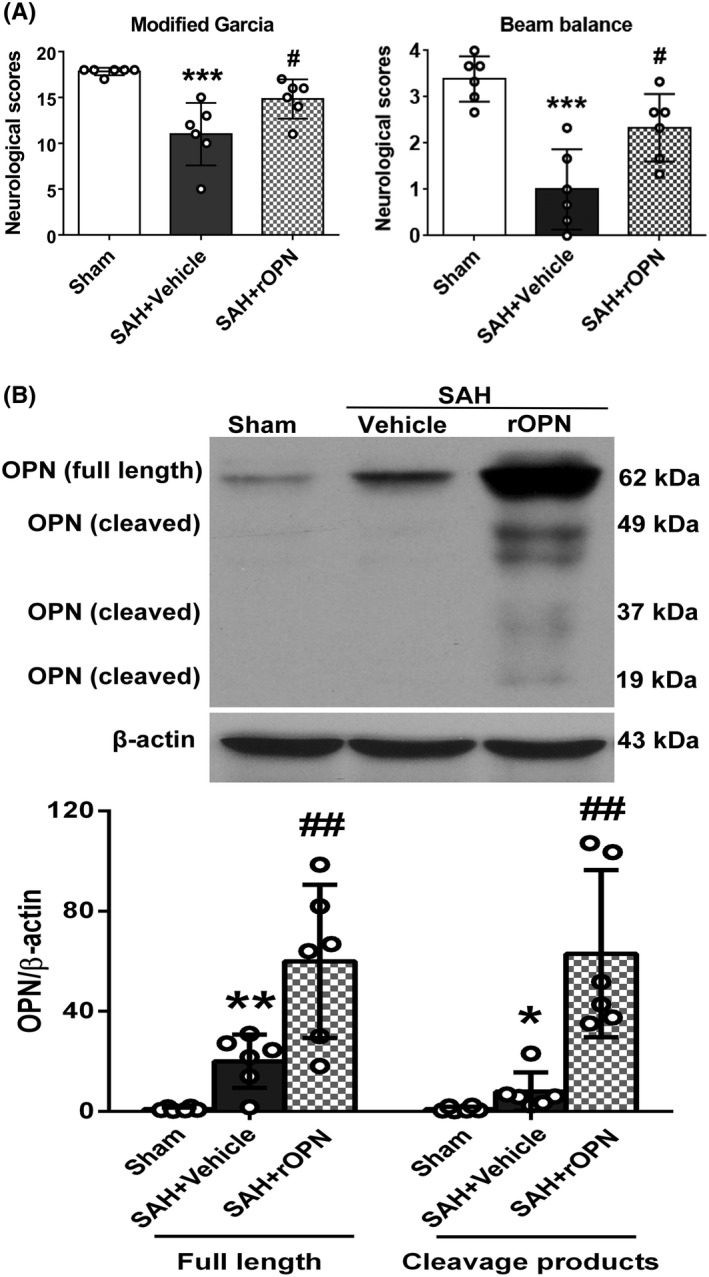
Intranasal administration of rat rOPN ameliorated neurological deficits at 24 h after SAH. A, rOPN improved modified Garcia score and beam balance score at 24 h after SAH. Sample size is 18, n = 6 per group. Data were analyzed using Kruskal‐Wallis test, For modified Garcia results, *χ* ^2^ = 13.441. For beam balance scores, *χ* ^2^ = 12.475. B, Intranasal administration of rat rOPN increased the total amount of full‐length OPN and its cleavage products in the left hemispheres of rat brain at 24 h after SAH. Sample size is 18, n = 6 per group. Data were presented as mean ± SD. For full‐length OPN, mean ± SD is 0.97 ± 0.6647 in Sham group, 20.08 ± 10.63 in SAH + Vehicle group, 59.93 ± 30.58 in SAH + rOPN group; *F* = 15.52. For OPN cleavage products, mean ± SD is 0.993 ± 0.9482 in Sham group, 7.963 ± 7.649 in SAH + Vehicle group, 62.98 ± 33.36 in SAH + rOPN group; *F* = 17.71. * P < .05, **P < .01, ***P < .001 vs Sham group; #*P* < .05, ##*P* < .01 vs SAH + Vehicle group

During the assessment of short‐term neurobehavior, modified Garcia and beam balance scores were significantly lower in the SAH + Vehicle group than those in the sham group (*P* < .001, Figure 3A). However, the intranasal administration of rOPN significantly improved neurological scores (SAH + rOPN group vs SAH + Vehicle group *P* < .05, Figure 3A).

### 3.5 rOPN administration elevated the total amount of OPN protein in the brain while suppressing apoptosis at 24 h after SAH

At 24 hours after SAH induction or sham surgery, Western blot analysis was performed to detect protein expressions after SAH and rOPN administration. Our results indicated that intranasal administration of rat rOPN drastically increased the level of full‐length OPN (62 kDa) and its cleavage products (20‐50 kDa) in the left hemisphere (SAH + rOPN group *vs.* SAH + Vehicle group *P* < .01, Figure 3B).

**Figure 4:**
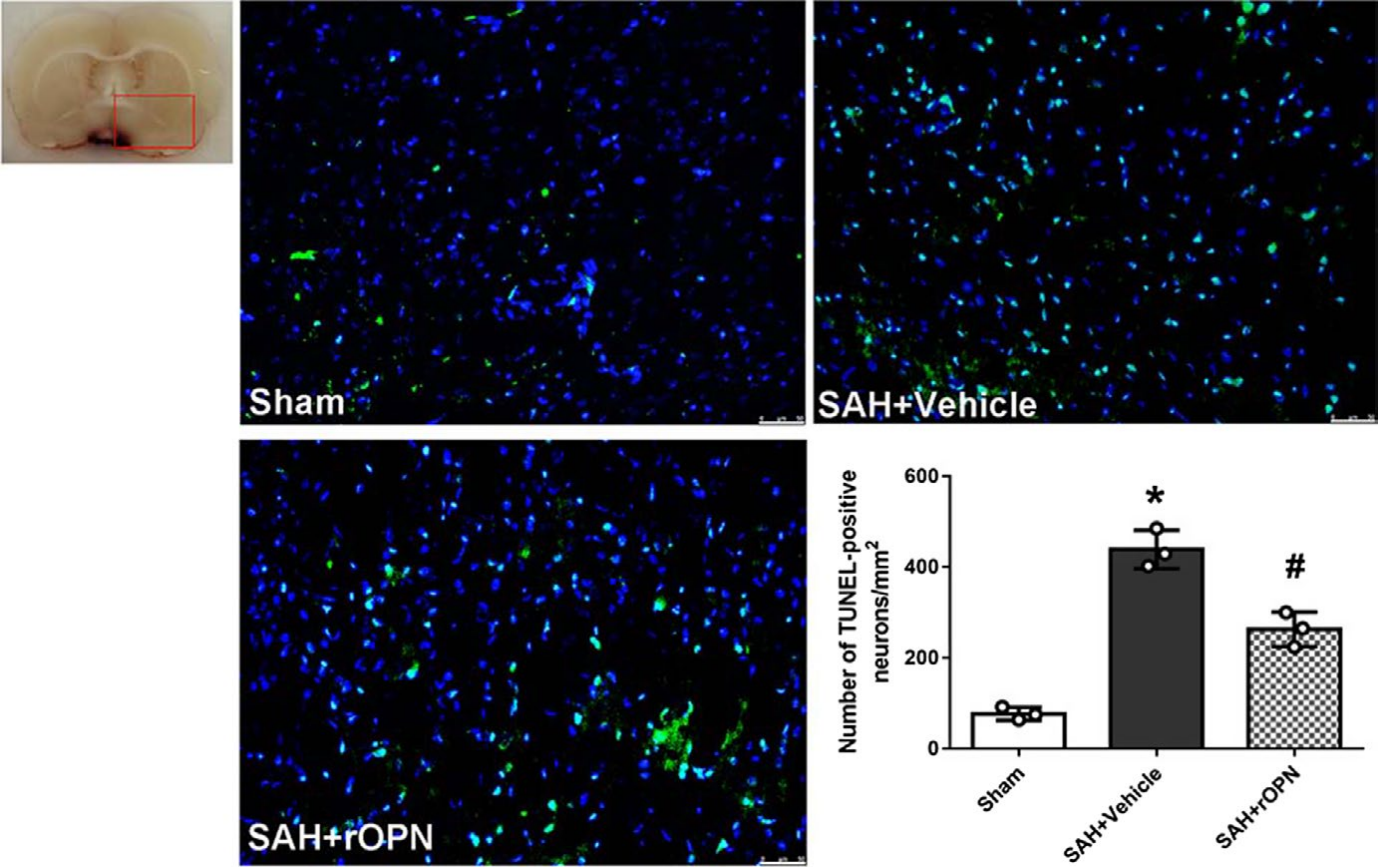
Intranasal administration of rat rOPN attenuated cell apoptosis in the brain at 24 h after SAH. rOPN administration significantly decreased the amount of TUNEL‐positive cells and attenuated cell apoptosis in the brain at 24 h after SAH. Sample size is 9, n = 3 per group. Data were presented as mean ± SD, mean ± SD is 77 ± 14.11 in Sham group, 438.7 ± 42.34 in SAH + Vehicle group, 263 ± 38.04 in SAH + rOPN group; *F* = 85.62. Scale bar = 50 µm. The red box on brain slice image indicates the locations observed. * *P* < .05 vs Sham group; #*P* < .05, vs SAH + Vehicle group

Moreover, TUNEL staining demonstrated there was a significant increase in the number of TUNEL‐positive cells at 24 hours after SAH when compared with the Sham group (*P* < .05, Figure 4). Also, rOPN administration remarkedly reduced the amount of TUNEL‐positive cells in the brain as compared with SAH + Vehicle group (*P* < .05, Figure 4).

### 3.6 rOPN administration elevated the expression of autophagy‐related proteins while suppressing apoptosis at 24 hours after SAH

**Figure 5:**
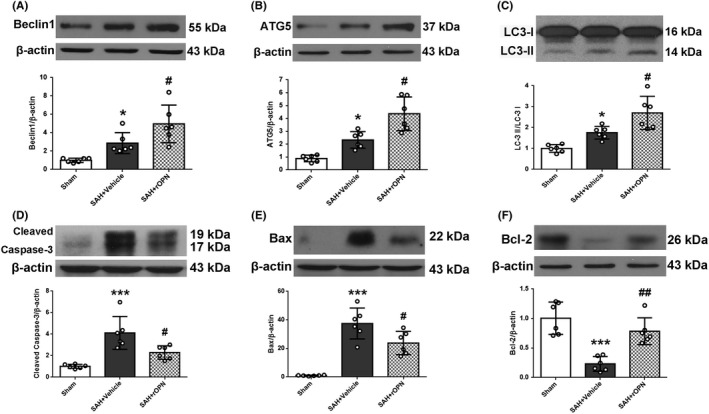
rOPN administration elevated the expression of autophagy‐related proteins while suppressing apoptosis in rat brain at 24 h after SAH. The effects of rOPN on expression levels of (A) Beclin 1, mean ± SD is 1.016 ± 0.2262 in Sham group, 2.874 ± 1.147 in SAH + Vehicle group, 4.963 ± 2.05 in SAH + rOPN group, *F* = 15.52, (B) ATG5, mean ± SD is 0.8908 ± 0.2545 in Sham group, 2.332 ± 0.6431 in SAH + Vehicle group, 4.364 ± 1.309 in SAH + rOPN group, *F* = 25.02, (C) LC3, mean ± SD is 1 ± 0.1845 in Sham group, 1.755 ± 0.3017 in SAH + Vehicle group, 2.7 ± 0.7957 in SAH + rOPN group, *F* = 17.23, (D) Cleaved Caspase‐3, mean ± SD is 1.008 ± 0.186 in Sham group, 4.112 ± 1.528 in SAH + Vehicle group, 2.291 ± 0.6268 in SAH + rOPN group, *F* = 15.86, (E) Bax, mean ± SD is 1.006 ± 0.321 in Sham group, 37.47 ± 10.86 in SAH + Vehicle group, 23.83 ± 8.143 in SAH + rOPN group, *F* = 33.13, (F) Bcl‐2, mean ± SD is 1.005 ± 0.2736 in Sham group, 0.2309 ± 0.1257 in SAH + Vehicle group, 0.7843 ± 0.2278 in SAH + rOPN group, *F* = 20.1, in the left hemisphere of rat brain at 24 h after SAH. Sample size is 18, n = 6 per group. Data were presented as mean ± SD. *P < .05, ***P < .001 vs Sham group; #*P* < .05, ##*P* < .01 vs SAH + Vehicle group

With the increase of OPN level in the rOPN‐treated group, the expression of Beclin 1, ATG5, and LC3 II to I ratio all increased, indicating enhanced autophagy level (SAH + rOPN group *vs.* SAH + Vehicle group *P* < .05, Figure 5 A, B and C). Meanwhile, the decrease of cleaved Caspase‐3 and Bax levels and an elevation of Bcl‐2 expression suggested attenuation of cell apoptosis (SAH + rOPN group *vs.* SAH + Vehicle group *P* < .05, Figure 5 D, E and F).

### 3.7 rOPN administration influenced the interaction and balance between autophagy and apoptosis at 24 hours after SAH

**Figure 6:**
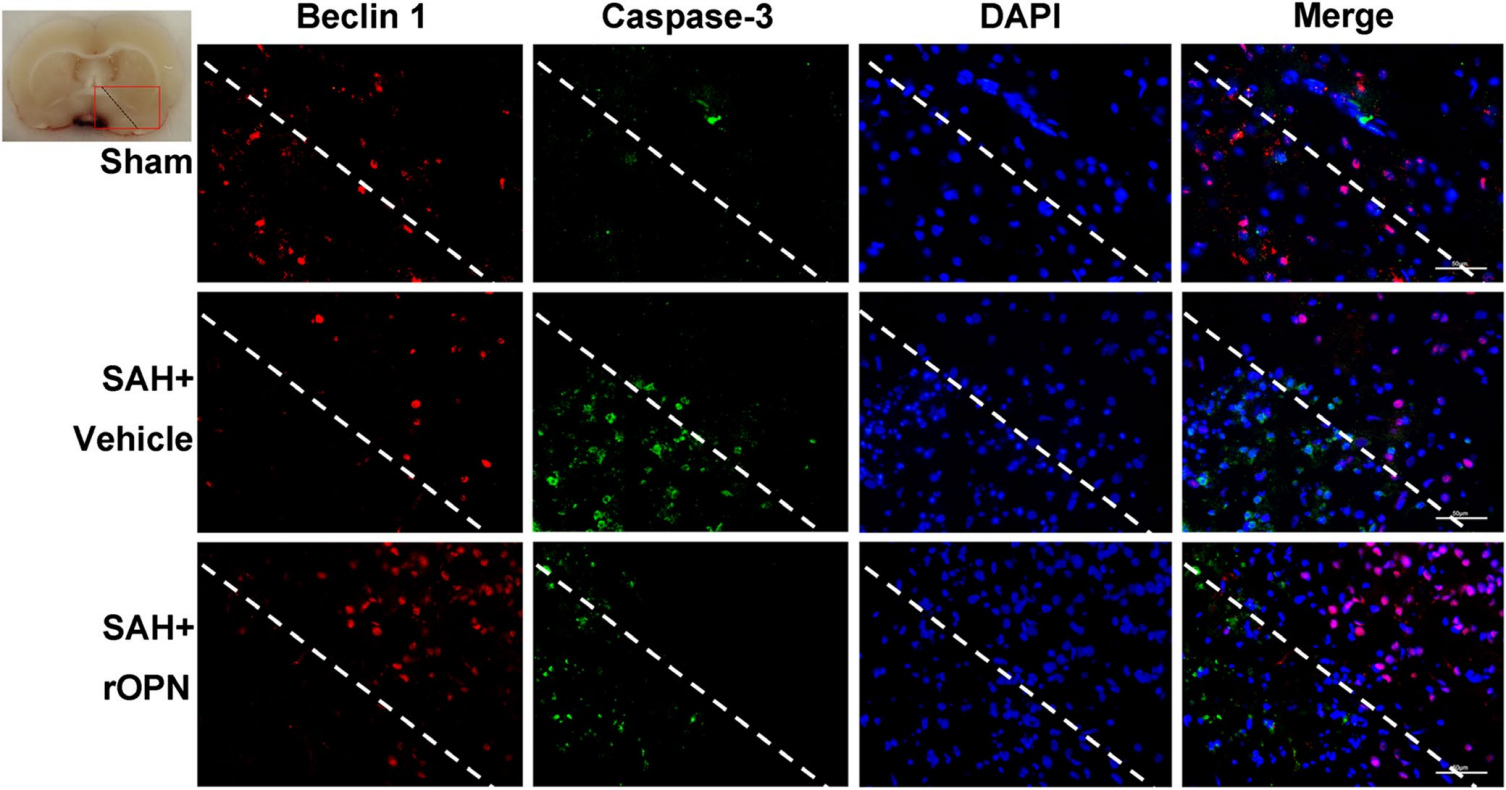
rOPN administration influenced the interaction and balance between Beclin 1 and Caspase‐3 at 24 h after SAH. Double immunofluorescence staining of Caspase‐3 and Beclin 1 in Sham group, SAH + Vehicle group and SAH + rOPN group at 24 h after SAH induction. Sample size is 9, n = 3 per group. Localization of Caspase‐3 can be cytoplasmic and nuclear. Staining in the nucleus is considered to be an indication of active Caspase‐3. The dashed lines and the red box on brain slice images indicate the locations observed. Vehicle, phosphate‐buffered saline; Scale bar = 50 μm

Based on our results above, we further performed double immunofluorescence staining of apoptosis marker Caspase‐3 with autophagy marker Beclin 1 to investigate the interaction of autophagy and apoptosis after SAH on the histological level (Figure 5). In the brain slices of sham rats, Beclin 1 and Caspase‐3 were both on relatively low expression levels. In both SAH + Vehicle and SAH + rOPN group, Caspase‐3 expression was mainly observed closely around the perforation site and blood clot, whereas Beclin 1 expression could only be observed at a little distance from the blood clot and not at the injured core. Moreover, Caspase‐3 and Beclin 1 expressions were not found in the same cell: when a cell expressed a high level of Beclin 1, it was Caspase‐3 negative and vice versa. Due to this unique distribution patterns of Caspase‐3‐positive cells and Beclin 1‐positive cells, we observed a “confrontation line” on the brain slice samples between green fluorescence cells (Caspase‐3 positive) and red fluorescence cells (Beclin 1‐positive) (Figure 5). This suggested that Caspase‐3 signaling and Beclin 1 signaling might be opposing signaling pathways for individual cells at 24 hours after SAH. Furthermore, in the comparison of SAH + Vehicle group and SAH + rOPN group, we found that administration of rOPN decreased the area with condensed Caspase‐3 staining while increasing the density of Beclin 1‐positive cells near the “confrontation line" (Figure 6).

## 4 DISCUSSIONS

Our main findings in the present study include: (a) all variants of endogenous OPN and autophagy‐related proteins (Beclin 1, ATG 5 and LC3 II to I ratio) increased after SAH and peaked at 24 hours; (b) major expression of OPN and Beclin 1 were found in neurons, indicating their neuroprotective roles; and (c) rOPN administration improved neurobehavior dysfunction, enhanced autophagy while suppressing apoptosis, and regulated autophagy‐apoptosis interaction.

OPN has been shown to have neuroprotective roles.^14^ Its biological functions are influenced by post‐translational modifications, including phosphorylation, glycosylation, and protein cleavage mediated by thrombin and metalloproteinases.^30^ Our previous studies reported that both intracerebroventricular and intranasal administration of rOPN had significant neuroprotective effects.^15^ Although intranasal administration has more clinical‐translational value,^15^ there remains no direct evidence for rOPN's post‐SAH delivery into the injured brain via the nasal route. Topkoru et al reported an increase of OPN levels in the cerebral spinal fluid of naïve rats after intranasal administration of rOPN. This indicated that rOPN could be delivered into the central nervous system via the nasal route.^21^ Gong et al reported that intranasal administration of rOPN dose‐dependently increased OPN expression in brain tissue after hyperglycemic intracerebralhemorrhage.^16^ In the current study, considering the reported importance of thrombin and metalloproteinases‐cleaved OPN after SAH,^31^, ^32^ we used Western blot analysis to examine the amount of full‐length OPN and cleaved OPN variants in the left hemisphere (the perforation side) after SAH and rOPN intranasal administration. Our results showed a significant increase in the amount of all variants of OPN, which includes both full‐length and cleavage products at 24 hours after SAH.

Autophagy is a highly dynamic process of cellular component degradation, which is often reflected by the conversion of LC3‐I to LC3‐II to form autophagosomes.^33^ Previous research demonstrated that autophagy has a neuroprotective role in EBI after SAH and that at least part of its protective role is through its anti‐apoptotic effect.^6^ Studies reported that specific therapeutic agents for SAH could activate cell autophagy while inhibiting apoptosis at the same time,^6^, ^10^, ^34^ which was consistent with the results in the current study. Furthermore, in the study of Shao et al, it was shown that 3‐MA inhibition of class III PI3K activity and autophagy activation^35^ led to increased cell apoptosis after SAH.^34^ In contrast, treatment with RAP, which induces autophagy by inhibiting mTOR,^36^ significantly decreases apoptosis level in the EBI phase.^9^ These previous results suggested that autophagy might be an upstream event to cell apoptosis after the onset of SAH.

However, these previous studies mainly focused on the quantification of autophagy and apoptosis‐related proteins using Western blot and TUNEL‐based cell count.^6^, ^9^, ^10^, ^34^ Few studies have investigated the interaction or balance between autophagy and apoptosis after SAH on the histological level. In a recent study by Guo et al,^12^ double immunofluorescence staining was performed with the autophagy marker p62 and apoptotic marker cleaved Caspase‐3 to show that resveratrol could upregulate autophagy (as indicated by the decreased expression level of p62), while inhibiting apoptosis (as indicated by the decreased expression level of cleaved Caspase‐3) following SAH in rats. However, the authors did not discuss whether p62 and Caspase‐3 could be coexpressed in the same cell, nor did they focus on the potential value of the autophagy‐apoptosis costaining results. In the current study, we performed double immunofluorescence staining of autophagy‐initiating protein Beclin 1 with apoptotic marker Caspase‐3 to observe their expression patterns and relationships on the histological level. First, we found the distribution patterns of the two markers differed after SAH: Caspase‐3 expression was condensed closely around the injured core (puncture site with blood clot), while Beclin 1 expression could only be observed at a little distance from the blood clot area but not at the injured core. Though there has been no in vivo study reporting the distribution patterns of autophagy and apoptosis proteins after SAH, one previous study in an in‐vitro model of ischemic cell injury did find that only a mild ischemic insult would lead to the induction of autophagy, while a moderate and severe insult primarily induced apoptosis and necrosis without autophagy.^11^ This finding was in line with the results in our current study, considering that in an endovascular perforation model of SAH severe damage and ischemic conditions are closer to the area around the perforation site and the blood clot. Furthermore, our results showed that 24 hours after SAH, cells were either stained with Caspase‐3 or with Beclin 1, but not both. This led us to the observation of a “confrontation line” between Caspase‐3‐positive cells and Beclin 1‐positive cells, suggesting that when a cell expressed a large amount of autophagy‐initiating protein Beclin 1, it would not enter late phase apoptosis (as indicated by Caspase‐3 negative). Thus, autophagy and apoptosis might be two opposing processes for individual cells at 24 hours after SAH. If combined with the conclusions of previous studies that autophagy was anti‐apoptotic and neuroprotective,^6^, ^10^, ^34^ then the results from our current study could be interpreted as after SAH, cells may face a choice between two different fates: autophagy (to live) or apoptosis (to die). With the administration of rOPN, the “confrontation line” could still be observed; however, the area with condensed Caspase‐3 expression decreased while the density of Beclin 1‐positive cells near the “confrontation line” increased as compared with vehicle samples. Our result was in line with a recent hypothesis that “Caspases may be the molecular switch node in the crosstalk between autophagy and apoptosis”.^37^

This study has several limitations. We only used Beclin 1 in immunofluorescence staining as the protein marker for autophagy due to limited experimental resources. However, while Beclin 1 is a crucial marker indicating the initiation of autophagy, LC3 punctae (which measures the signals of LC3‐II) is a more accurate way to reflect the level of autophagy in histological studies.^10^ In the current study, we tried to control that the area photographed in all the samples (throughout all three groups, with three rats in each group) stay as similar as possible. Still, future studies should include methods such as stereological quantification for a more accurate and thorough observation, or even quantification, of the “autophagy‐apoptosis costaining” phenomenon.^38^, ^39^ Furthermore, ferroptosis, which is a newly described form of programmed cell death (PCD) that is dependent on reactive oxygen species and iron, has been demonstrated to play crucial roles in the pathophysiology of hemorrhagic stroke including SAH.^40^, ^41^ There have been no studies investigating whether administration of rOPN has any effect on post‐SAH ferroptosis, and it remains to be elucidated in further studies.

## 5 CONCLUSION

In conclusion, rOPN administration could activate autophagy while attenuating apoptosis at 24 hours after SAH through regulating the interaction between Caspase‐3 and Beclin 1. Further investigations of the relationship between autophagy and apoptosis on the histological level using the “autophagy‐apoptosis costaining method” described in the current study are needed to better understand precise molecular mechanisms of the interactions between autophagy and apoptosis in the brain cells after SAH. Future studies should also consider how therapeutic agents such as OPN can influence this autophagy‐apoptosis interaction to exert their neuroprotective effects.

## CONFLICT OF INTEREST

The authors declare no conflict of interest.
